# ^18^F-FDG PET, cognitive functioning, and CSF biomarkers in patients with obstructive sleep apnoea before and after continuous positive airway pressure treatment

**DOI:** 10.1007/s00415-022-11182-z

**Published:** 2022-05-24

**Authors:** Mariana Fernandes, Luisa Mari, Agostino Chiaravalloti, Barbara Paoli, Marzia Nuccetelli, Francesca Izzi, Maria Pia Giambrone, Riccardo Camedda, Sergio Bernardini, Orazio Schillaci, Nicola Biagio Mercuri, Fabio Placidi, Claudio Liguori

**Affiliations:** 1 Department of Systems Medicine, Sleep Medicine Centre, University of Rome “Tor Vergata”, Rome, Italy; 2 Neurology Unit, University Hospital of Rome “Tor Vergata”, Rome, Italy; 3 Department of Biomedicine and Prevention, University of Rome “Tor Vergata”, Rome, Italy; 4 IRCCS Neuromed, Pozzilli, Italy; 5 Department of Clinical Biochemistry and Molecular Biology, University of Rome “Tor Vergata”, Rome, Italy; 6 IRCSS Santa Lucia Foundation, Rome, Italy

**Keywords:** Obstructive sleep apnoea, Positron emission tomography, Cognition, Cerebrospinal fluid, Continuous positive airway pressure

## Abstract

**Introduction:**

Dysregulation of cerebral glucose consumption, alterations in cerebrospinal fluid (CSF) biomarkers, and cognitive impairment have been reported in patients with obstructive sleep apnoea (OSA). On these bases, OSA has been considered a risk factor for Alzheimer’s disease (AD). This study aimed to measure cognitive performance, CSF biomarkers, and cerebral glucose consumption in OSA patients and to evaluate the effects of continuous positive airway pressure (CPAP) treatment on these biomarkers over a 12-month period.

**Methods:**

Thirty-four OSA patients and 34 controls underwent ^18^F-fluoro-2-deoxy-d-glucose positron emission tomography (^18^F-FDG PET), cognitive evaluation, and CSF analysis. A subgroup of 12 OSA patients treated with beneficial CPAP and performing the 12-month follow-up was included in the longitudinal analysis, and cognitive evaluation and ^18^F-FDG PET were repeated.

**Results:**

Significantly reduced glucose consumption was observed in the bilateral praecuneus, posterior cingulate cortex, and frontal areas in OSA patients than controls. At baseline, OSA patients also showed lower β-amyloid_42_ and higher phosphorylated-tau CSF levels than controls. Increased total tau and phosphorylated tau levels correlated with a reduction in brain glucose consumption in a cluster of different brain areas. In the longitudinal analysis, OSA patients showed an improvement in cognition and a global increase in cerebral ^18^F-FDG uptake.

**Conclusions:**

Cognitive impairment, reduced cerebral glucose consumption, and alterations in CSF biomarkers were observed in OSA patients, which may reinforce the hypothesis of AD neurodegenerative processes triggered by OSA. Notably, cognition and brain glucose consumption improved after beneficial CPAP treatment. Further studies are needed to evaluate the long-term effects of CPAP treatment on these AD biomarkers.

## Introduction

Obstructive sleep apnoea (OSA) is the most frequent sleep-disordered breathing (SDB) and is highly prevalent in middle-aged and older adults [1]. OSA is characterised by repetitive episodes of upper airway obstruction, leading to apnoea and hypopnoea events, intermittent hypoxia, and sleep fragmentation [2, 3]. OSA is a risk factor for several morbidities [1, 4–8] and is associated with the risk of cognitive impairment [9]. Recent evidence suggests that OSA may also be a risk factor for Alzheimer’s disease (AD) neurodegeneration [10–13], given that it alters cerebral β-amyloid metabolism and promotes neuroinflammation and oxidative stress [3, 10–19]. However, in contrast to other proven risk factors for the development of AD, OSA can be treated in clinical practice through the use of continuous positive airway pressure (CPAP), which significantly improves OSA symptoms [20].

Decreased cerebrospinal fluid (CSF) levels or documentation of plaque deposition of β-amyloid_42_ (Aβ_42_), reduced cortical temporo-parietal ^18^F-fluoro-2-deoxy-d-glucose uptake (^18^F-FDG) on positron emission tomography (PET), and increased CSF levels of total-tau (t-tau) and phosphorylated-tau (p-tau) have been considered biomarkers of AD neuropathology and are currently used to support AD diagnosis in both clinical practice and research [21–24]. These biomarker alterations appear early during the course of the AD pathology, usually before brain magnetic resonance imaging (MRI) structural changes. Accordingly, they have been validated in providing early high diagnostic accuracy during the work-up for AD [25]. In the recent past, pathological modifications of CSF biomarkers have already been described in patients with OSA, with or without subtle cognitive impairment [10, 14–16, 26–29]. Conversely, fewer ^18^F-FDG PET brain studies have been performed in patients with OSA and included very small groups of patients. These studies documented a significant modification of ^18^F-FDG uptake in different brain areas, in particular, glucose hypometabolism in the bilateral prefrontal areas, praecuneus, left hippocampus, and left anterior cingulate cortex, among others [30–32]. One of the more recent studies reported that despite the presence of subtle memory impairment, OSA patients displayed a significant reduction in cerebral glucose consumption in the praecuneus, cingulate, parieto-occipital, and prefrontal cortices [33]. However, studies evaluating brain glucose consumption using ^18^F-FDG PET in OSA patients remain limited, and the possible effects of prolonged CPAP treatment were not evaluated, since the longitudinal studies were set at a 3-month follow-up [30–33]. Finally, previous studies evaluating patients with OSA analysed CSF and PET data obtained weeks or months after the diagnosis of OSA. Therefore, the present study aimed to comprehensively measure cognition, CSF biomarkers, and cerebral glucose uptake by ^18^F-FDG PET in middle-aged adult patients with moderate to severe OSA (apnoea–hypopnoea index [AHI] ≥ 15/h) during the diagnostic work-up of the sleep disorder. A subgroup of patients was also followed for a period of 12 months and repeated cognitive testing and brain ^18^F-FDG PET. Moreover, to better understand the direction of the biomarker changes obtained at baseline, associations between the measured CSF biomarkers of AD pathology (tau proteins and Aβ_42_), cerebral glucose uptake and cognitive performance were analysed.

## Methods

### Participants and study procedures

**Fig. 1:**
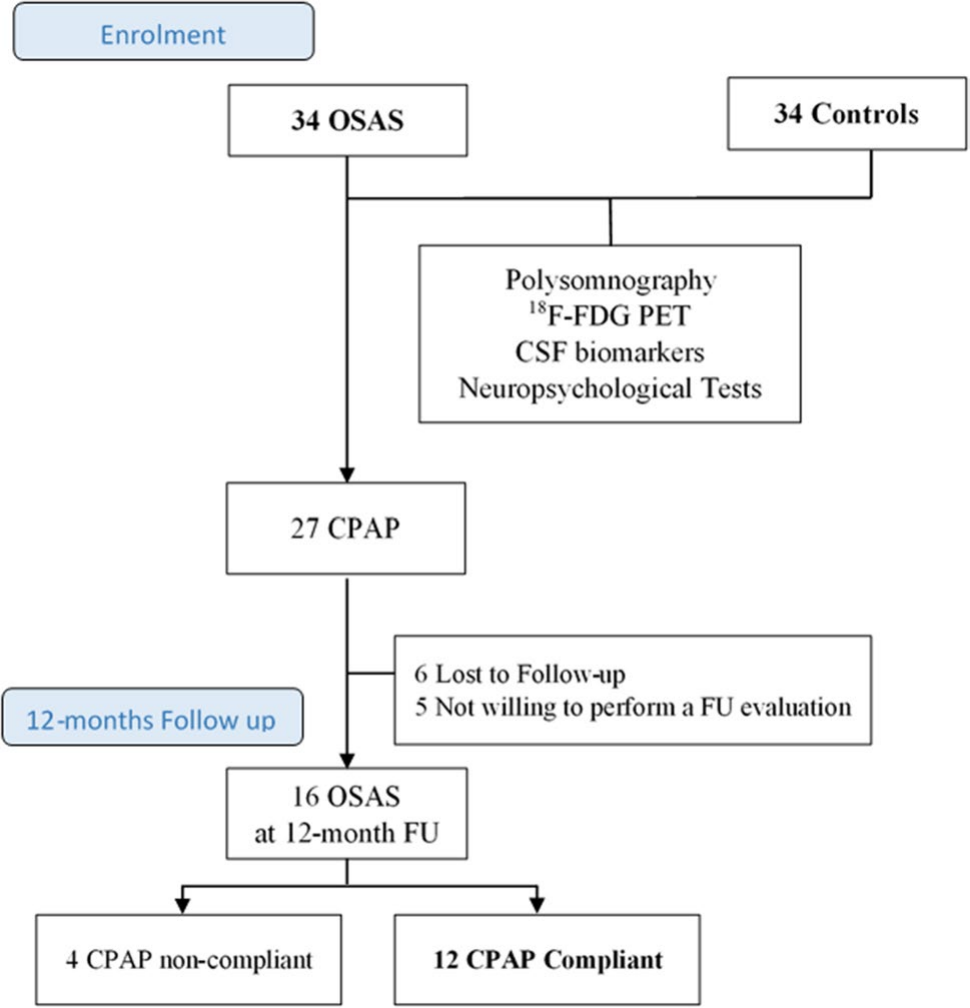
Flow chart of the recruitment and selection process

Middle-aged adult patients with OSA who were admitted to the Sleep Medicine Centre at the Neurology Unit of the University Hospital of Rome “Tor Vergata” were recruited between January 2016 and September 2018. All patients underwent a standard sleep medicine visit, polysomnographic recording (PSG), physical and neurological examinations, cognitive evaluation, CSF AD biomarker analysis, and ^18^F-FDG PET. A subgroup of patients with moderate to severe OSA (AHI ≥ 15/h), compliant with CPAP treatment for a period of 12 months, were included in the longitudinal analysis to evaluate changes in neuropsychological and neuroimaging biomarkers. At follow-up, the patients underwent cognitive evaluation and ^18^F-FDG PET. Figure 1 shows a flowchart of the recruitment and selection process.

The inclusion criteria for OSA patients were as follows: no concomitant neurological or psychiatric diseases, no dementia (Clinical Dementia Rating [CDR] > 1), no concomitant other sleep disorders, and no use of CNS-active drugs. Participants with systemic and/or neurologic infectious, inflammatory, or autoimmune diseases; diabetes; clinical conditions influencing cognitive performance (such as hypothyroidism or B12 vitamin depletion); and history of alcohol or other substance abuse were excluded from the present study. Moreover, brain MRI was used to exclude signs of brain atrophy, particularly in the hippocampus, and white matter abnormalities due to stroke or asymptomatic vessel disease. Inclusion criteria for the longitudinal analysis were as follows: good compliance to CPAP treatment (as previously defined as greater than 4 h of CPAP use per night on a routine basis [> 5 nights per week]) [34–36] and a mean residual of AHI, obtained by the ventilator software report, < 10 per hour.

A group of controls was also recruited at the University Hospital of Rome “Tor Vergata”. Specifically, the control group (CG) included inpatients at the same medical centre undergoing clinical neurologic examination and ^18^F-FDG PET for suspected malignancies, which were ruled out after diagnostic investigations. All the controls underwent physical and neurological examinations, cognitive evaluation, lumbar puncture (LP), and ^18^F-FDG PET. Moreover, a sleep medicine interview to exclude sleep disorders was performed in the controls. Exclusion criteria were signs or symptoms of central or peripheral nervous system diseases, diabetes, depression or other psychiatric symptoms, cognitive decline, and sleep disorders.

The study was performed according to the STROBE statement, and the study protocol was considered as observational and approved by the Ethical Committee of the University Hospital of Rome “Tor Vergata”. Written informed consent was obtained from all participants in the study, and subjects and procedures were followed in accordance with the ethical standards of the responsible committee on human experimentation (institutional and national) and with the Helsinki Declaration of 1975, as revised in 2013 [37].

### Polysomnographic recordings

All OSA patients underwent PSG recording to evaluate nocturnal sleep (SOMNOscreen, SOMNOmedics GmbH, Randersacker, Germany). The following standard parameters were computed: sleep onset latency (SL; the time-interval between the lights off and the first sleep epoch), total sleep time (TST; the actual sleep time without SL and awakenings), sleep efficiency (SE; the ratio between TST and time in bed), rapid eye movement (REM) sleep latency (REML; the time interval between the sleep onset and the first epoch of REM sleep), stage 1 of non-REM sleep (N1), stage 2 of non-REM sleep (N2), stage 3 of non-REM sleep (N3), REM sleep (REM), and wakefulness after sleep onset (WASO). Sleep stages were calculated as percentages of the TST. Apnoea was defined as a reduction of > 90% of respiratory airflow for 10 or more seconds, while hypopnoea has been determined as the reduction of  > 30% of respiratory airflow for 10 or more seconds associated with an oxygen desaturation of  ≥ 3%. The severity of OSA is determined by AHI (the sum of all apnoeas and hypopnoeas per hour of sleep). The following oxygen saturation (*Sa*O_2_) parameters were evaluated: mean *Sa*O_2_, nadir *Sa*O_2_, time spent with *Sa*O_2_ < 90% (*T* < 90), and ODI (number of oxygen desaturations ≥ 3% per hour). PSG recordings were scored based on the international standard criteria of the American Academy of Sleep Medicine [38].

### Neuropsychological tests

Cognitive evaluation was performed the day after PSG recording, immediately before performing LP. The Mini-Mental State Examination (MMSE) [39] and a brief revised version of the Mental Deterioration Battery [40, 41] were performed in all participants. All scores were corrected for age and education level. Short- and long-term memory, executive function, attention, and general intelligence were evaluated [26].

Short- and long-term memory was evaluated using the Rey Auditory-Verbal Learning Test (RAVLT) [42]. A list of 15 words was read to the patient five times and their ability to recall was measured immediately (the sum of the words recalled in the five trials, RAVLT-I) and after 15 min (the number of words recalled 15 min after the last word presentation, RAVLT-D).

Executive functions were evaluated using the Stroop Colour/Word test in which the participants were required to name the colour ink that a colour-word (e.g. RED) is presented in, both in congruent (e.g. when the word RED is printed in red link) and incongruent conditions (e.g. when the word BLUE is printed in red link). In the latter condition, an increase in the number of errors and the time taken to respond is observed (“Stroop interference effect”) [43]. The test is considered the “paradigmatic measure of selective attention” [44].

General intelligence was tested using Raven Progressive Matrices (36 items), which consists of choosing from a set of distractors the item logically missing in a given visual/spatial set [45]. The items are regrouped in a set of three subtests (labelled A, Ab, and B) and evaluate non-verbal intelligence, visual processing speed, cognitive speed, and flexibility.

### CSF biomarker analysis

All CSF samples were obtained the day after the PSG recording by LP performed in the decubitus position between 8:00 and 9:30 AM, within 1–2 h after morning awakening, using an atraumatic needle. CSF samples were collected in polypropylene tubes using standard sterile techniques. The first 4 mL CSF sample was used for routine biochemistry analysis, including the total cell count. A second 4 mL CSF sample was centrifuged to eliminate cells and cellular debris and immediately frozen at − 80 °C until the analysis to assess t-tau, p-tau, and Aβ_42_ levels could be performed. The CSF Aβ_42_, t-tau, and p-tau levels were determined according to previously published standard procedures, using commercially available sandwich enzyme-linked immunosorbent assays (Innotest β-Amyloid 1–42, Innotest h-T-tau, Innotest Phospho-T-tau 181; Innogenetics, Ghent, Belgium) [15, 46–48]. The cut-off values for the CSF biomarkers positive for AD pathology were set in-house [49, 50], and our laboratory results are in line with those of the external quality control program for the CSF biomarkers, promoted by the Alzheimer’s Association [51]. Specifically, Aβ_42_, t-tau, and p-tau were dichotomized on the basis of previously established cut-off values: < 500 pg/mL for Aβ_42_, > 375 pg/mL for t-tau, and > 52 pg/mL for p-tau [46, 48].

### PET/CT scanning protocol

The PET/CT protocol study was conducted at the Nuclear Medicine facility of the University Hospital of Rome “Tor Vergata”. All subjects, the day before performing PSG, were intravenously injected with ^18^F-FDG (dose range 185–295 megabequerels) and hydrated with 500 mL of saline (0.9% sodium chloride). PET/CT acquisition, using a General Electric VCT PET/CT scanner, started 30 ± 5 min after ^18^F-FDG injection and lasted 10 min for all participants. The reconstruction parameters were as follows: ordered subset expectation maximisation, 4 subsets and 14 iterations; matrix 256 × 256; full width at half maximum (FWHM): 5 mm [52].

## Data and statistical analysis

### PSG, neuropsychological, and CSF data analysis

A commercial statistical software was used for statistical analysis (SPSS version 25, IBM Corporation, Armonk, NY, USA) [53]. The Kolmogorov–Smirnov test was used to check for normal distribution of PSG, neuropsychological, and CSF data. All demographic, clinical, PSG, neuropsychological, and CSF data were then compared between the two groups (OSA vs. CG) using the Student’s t-test. For the subgroup analyses, considering the small sample size, differences between clinical and cognitive functioning of patients at baseline and at the 12-month follow-up were tested using the Wilcoxon signed rank test.

Correlations between the CSF biomarker levels, neuropsychological, ^18^F-FDG PET, and PSG data in OSA patients were performed using Pearson’s correlation test. Only the statistically different ^18^F-FDG PET areas between the OSA and CG were included in the Pearson correlations. To reduce the chances of obtaining false-positive results, due to the multiple comparisons among the demographic, clinical, sleep parameters and neuropsychological scores, a Bonferroni correction was used. The Bonferroni correction divides the unadjusted *p* values by the total number of tests, which corresponds to an alpha level of 0.003 (0.05/20). All p-values lower or equal to 0.003 were considered as statistically significant. Effect sizes were quantified by Cohen’s *d*.

### ^*18*^*F-FDG PET analysis*

Statistical parametric mapping 12 (SPM12) implemented in MATLAB 2018a was used to analyse PET scans in this study (https://www.fil.ion.ucl.ac.uk/spm/software/spm12/), as previously reported [54]. PET data were converted from DICOM to NIfTI format using MRIcro software available at https://www.nitrc.org/projects/mricron and then subjected to a normalisation process. A bias regularisation was applied (0.0001) to limit biases due to smooth, spatially varying artefacts that modulate the image’s intensity and impede the automated processing of the images. To prevent the algorithm from trying to model the intensity variation due to different tissue types, the FWHM of the Gaussian smoothness of bias was set at a 60-mm cut-off. A tissue probability map implemented in SPM12 was used (TPM.nii). To achieve approximate alignment to the ICBM space template—European brains [55, 56], a mutual information affine registration was used with the tissue probability maps [57].

Warping regularisation was set with the following 1 × 5 arrays (0, 0.001, 0.5, 0.05, 0.2). To cope with functional anatomical variability that is not compensated by spatial normalisation and improves the signal-to-noise ratio, smoothness was set at 5 mm, and the sampling distance, which encodes the approximate distance between sampled points when estimating the model parameters, was set at 3.

To blur the individual variations (especially gyral variations) and to increase the signal-to-noise ratio, an 8-mm isotropic Gaussian filter was applied. Before regression analysis was applied, it was necessary to define the parameters and post-processing tools; global normalisation (which escalates images to a global value) = 50 (using proportional scaling); the masking threshold (to help identify voxels with an acceptable signal in them) was set to 0.8; a transformation tool of statistical parametric maps into normal distribution was used; the correction of SPM coordinates to match the Talairach coordinates, subroutine implemented by Matthew Brett (http://www.mrc-cbu.cam.ac.uk/Imaging) was made. Brodmann areas (BA) were identified at a range from 0 to 3 mm from the corrected Talairach coordinates of the SPM output isocentre using a Talairach client available at http://www.talairach.org/index.html. As proposed by Bennett et al*.* [58], SPM t-maps were corrected for multiple comparisons using the false discovery rate (*P* ≤ 0.05) and corrected for multiple comparisons at the cluster level (*P* ≤ 0.001). The level of significance was set at 100 (5 × 5 × 5 voxels, i.e. 11 × 11 × 11 mm) contiguous voxels [58]. The following voxel-based comparisons were assessed: (1) CG versus OSA patients at baseline and vice versa; (2) OSA patients at baseline vs. OSA patients after CPAP therapy and vice versa. In order to exclude possible abnormalities or biases due to the presence of a very early AD stage in the subgroup of OSA patients, a separate analysis was performed, comparing patients with normal CSF biomarkers to patients showing the CSF AD biomarkers consistent with possible AD pathology (low Aβ_42_ and/or high t-tau or p-tau). Moreover, a single subject analysis was performed to identify the presence of typical AD ^18^F-FDG PET patterns at baseline [59]. All comparisons were performed using the “two-sample t-test” design model available in SPM12 [52]. Sex and MMSE were used as covariates in the analyses of the CG and OSA patients.

To investigate the relationship between ^18^F-FDG brain uptake and neuropsychological testing in OSA subjects, a selected ROI was placed on the cortical grey matter of Bas found to be affected by a relative hypometabolism in OSA patient vs. control comparisons using the WFU Pickatlas tool (https://www.nitrc.org/projects/wfu_pickatlas/) implemented in SPM 12 and further analysed after a normalisation process [60]. The mean signal intensities computed for the whole cluster were normalised within each subject to the average intensities of the cerebellar volume of interest, as defined by other reports published previously [60].

## Results

### Demographic and clinical data

Thirty-four patients affected by moderate to severe OSA were recruited for this study, and the same number of subjects was included in the CG. Demographic and clinical data of the study groups are reported in Table 1, and the PSG results and neuropsychological data of OSA patients are presented in Table 2. According to the neuropsychological data, OSA patients showed lower MMSE scores (25.87 ± 2.63) than controls (29.08 ± 0.90) (*p* = 0.001, *d* = 1.63).

**Table 1:** Demographic and clinical data of OSA patients and controls

Demographic and clinical data	OSA (*n* = 34) (mean ± SD)	Controls (*n* = 34) (mean ± SD)	*p* value*
Age (years)	68.44 ± 9.98	69.02 ± 6.08	NS
Gender	27 M/ 7 F	27 M/ 7 F	NS
Education (years)	9.17 ± 3.56	10.51 ± 2.87	NS
ApoE4	6/34	4/34	NS
**Neuropsychological data**			
MMSE p.v. < 24*	25.87 ± 2.63 7/34	28.08 ± 0.9 0/34	0.001
CDR	0.19 ± 0.25	NA	NA
*1 patient with MMSE = 23 and 6 patients with MMSE = 22			
**CSF data**			
Aß_42_ (pg/mL) p.v. < 500 pg/mL	622.78 ± 211.96 7/34	921.96 ± 69.55 0/34	0.002
t-tau (pg/mL) p.v. > 375 pg/mL	335.30 ± 335.39 6/34	252.89 ± 43.26 0/34	NS
p-tau (pg/mL) p.v. > 52 pg/mL	62.34 ± 49.37 9/34	32.75 ± 5.21 0/34	0.001

*Abbreviations: OSA* obstructive sleep apnoea, *M* male, *F* female, *ApoE4* apolipoprotein epsilon 4, *MMSE* Mini-Mental State Examination, *CDR* clinical dementia rating scale, *Aß*_*42*_ ß-amyloid_42_, *t-tau* total tau, *p-tau* phosphorylated tau, *p.v.* pathological value, *SD* standard deviation, *NA* not applied, *NS* not significant

*Significance is set as *p* < 0.003 (Bonferroni corrected alpha)

**Table 2:** Polysomnographic and cognitive data of OSA patients

	OSA patients (*n* = 34) (mean ± SD)
**PSG data**	
TST (min)	347 ± 71.86
SE (%)	77.53 ± 12.34
SL (min)	7.1 ± 6.16
REML (min)	121.37 ± 68.37
% REM	13.14 ± 5.92
% N1	13.7 ± 8.68
% N2	57.70 ± 13.00
% N3	18.05 ± 9.08
WASO (min)	100.59 ± 57.07
AHI	44.52 ± 20.46
*Sp*O_2_ nadir *(*%*)*	79.95 ± 5.94
Mean *Sp*O_2_ *(*%*)*	94.37 ± 1.85
Time with *s*O_2_ < 90% (min)	10.19 ± 12.64
ODI	34.8 ± 16.69
**Cognitive data**	
RAVLT-I (p.v. < 28.53)	27.52 ± 10.28 17/34
RAVLT-D (p.v. < 4.69)	5.04 ± 3.17 13/34
Raven (p.v. < 18.96)	26.61 ± 7.16 5/34
Stroop (T) (p.v. > 31.66)	42.85 ± 28.82 13/34
Stroop (E) (p.v. > 2.83)	2.35 ± 2.56 10/34

*Abbreviations: TST* total sleep time, *SE* sleep efficiency, *SL* sleep latency, *REML* REM latency, *N1* stage 1 of non-REM sleep, *N2* stage 2 of non-REM sleep, *N3* stage 3 of non-REM sleep, *WASO* wake after sleep onset, *AHI* apnoea-hypopnoea index, *sO*_*2*_ oxygen saturation, *ODI* oxygen desaturation index, *RAVLT-I* Rey auditory verbal learning test, immediate recall, *RAVLT-D* Rey auditory verbal learning test, delayed recall, *T* time, *E* error

### CSF data

OSA patients showed lower CSF Aβ_42_ levels than controls (*p* = 0.002, *d* = 1.90), with 7 OSA patients (20.5% of the sample) presenting pathologic Aβ_42_ values (below cut-off < 500 pg/mL). OSA patients also showed higher CSF levels of p-tau *(p* = 0.001,* d* = 0.84*)* when compared to controls. Moreover, six OSA patients (17.6% of the sample) showed pathological t-tau CSF levels (above cut-off > 375 pg/mL) and 9 patients (26.4%) showed pathological p-tau CSF levels (above cut-off > 52 pg/mL). No AD biomarker CSF values were pathological in the CG. All CSF data for patients with OSA and controls are reported in Table 1.

### ^18^F-FDG PET analysis

**Fig. 2:**
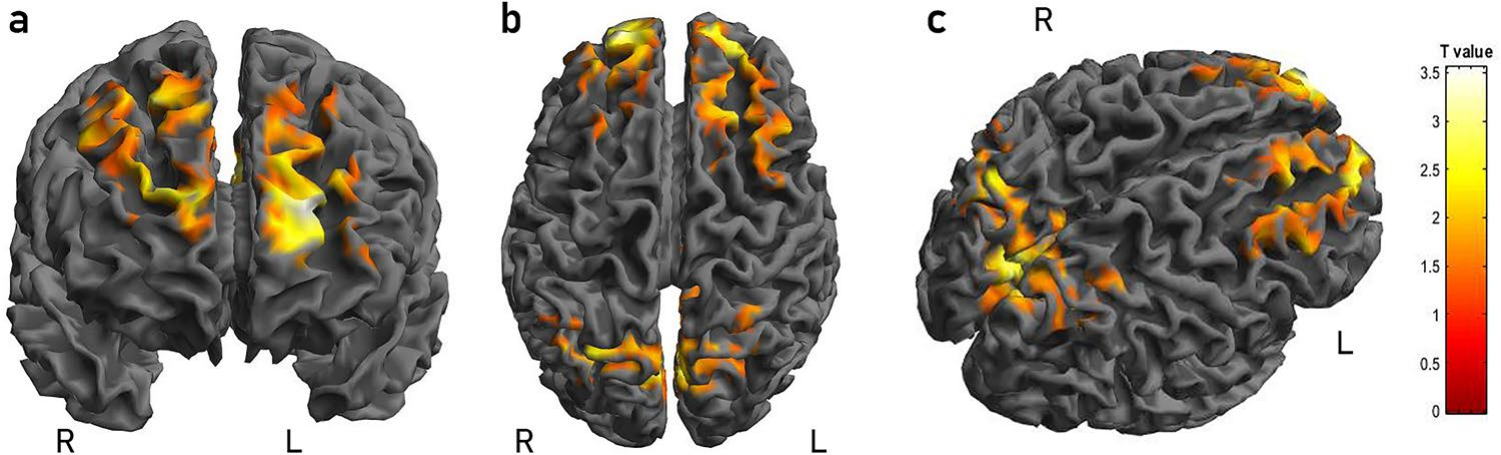
Frontal (**a**), upper (**b**) and lateral (**c**) view of the three-dimensional (3D) rendering showing the results of SPM comparisons between 18 F-FDG uptake in OSA patients as compared to the control group. The significance values above a chosen threshold and the “T value” in this voxel for a given contrast is represented by use of a colour intensity code. OSA patients show a significant reduction of brain glucose consumption in left and right frontal, parietal and in right limbic cortex. Details are provided in Table 3. R: right; L: left

Considering ^18^F-FDG PET analysis, a reduction in ^18^F-FDG PET uptake was found in patients with OSA in the bilateral praecuneus (BA 7), posterior cingulate cortex (BA 23), and frontal areas (BA8-9-10) when compared to the CG (Table 3 and Fig. 2). No significant differences were found when comparing patients with normal CSF AD biomarkers to patients with CSF AD biomarkers alterations. Moreover, no significant results were obtained in the single-subject analyses.

**Table 3:** Numerical results of SPM comparisons between ^18^F-FDG uptake in OSA vs. controls

Analysis	Cluster level					Voxel level		
	Cluster p(FWE-corr)	Cluster p(FDR-corr)	Cluster extent	Cortical Region	Z score of maximum	Talairach coordinates	Cortical region	BA
OSA—Controls	0.037	0.0178	1414	L frontal	4.51	− 22.60.16	Superior frontal gyrus	10
				L frontal	3.34	− 14.46.38	Superior frontal gyrus	8
				L frontal	3.21	− 30.44.34	Middle frontal gyrus	9
	< 0.0001	< 0.0001	4705	R parietal	4.16	2.− 72.34	Precuneus	7
				L parietal	4.05	− 8.− 72.34	Precuneus	7
				R limbic	3.97	4.− 58.16	Posterior cingulate	23
	0.011	0.008	1833	R frontal	4.14	26.38.46	Superior frontal gyrus	8
				R frontal	3.86	22.56.24	Superior frontal gyrus	10
				R frontal	3.61	14.60.20	Superior frontal gyrus	10

In the “cluster level” section on the left, the number of voxels, the corrected *P* value of significance, and the cortical region where the voxel is found, are all reported for each significant cluster. In the “voxel level” section, all of the coordinates of the correlation sites (with the Z-score of the maximum correlation point), the corresponding cortical region and BA are reported for each significant cluster. L, left; R, right; BA, Brodmann area. When the maximum correlation is achieved outside the grey matter, the nearest grey matter (within a range of 5 mm) is indicated by the corresponding BA

### Correlations analysis

In the OSA patient group, a significant correlation was found between ^18^F-FDG PET and CSF biomarkers, indicating that a reduction in ^18^F-FDG PET cerebral uptake was associated with higher t-tau and p-tau CSF concentrations. No significant correlations were observed between the CSF levels and cognitive performance. Table 4 shows the significant correlations among ^18^F-FDG PET, demographic characteristics, CSF biomarkers, and cognitive data.

**Table 4:** Pearson correlations among ^18^F-FDG uptake as detectable in PET,  demographic characteristics, CSF biomarkers, and cognitive data

	AHI	LBA7	LBA8	LBA9	LBA10	RBA7	RBA8	RBA10	RBA23
Age									
Education									
Aβ42									
t-tau		− 0.69*	− 0.60*	− 0.63*	− 0.62*	− 0.660*	− 0.70*	− 0.62*	
p-tau		− 0.54*			− 0.61*	− 0.614*	− 0.66*	− 0.61*	
MMSE									
RAVLT-I									
RAVLT-D									
Raven Progressive Matrices									
Stroop (T)									
Stroop (E)					^*^				

*AHI* apnoea–hypopnoea index, *LBA7* left precuneus level (7 Broadman area), *LBA8-10* left frontal areas (8-9-10 Broadman area), *RBA7* right precuneus level (7 Broadman area), *RBA8 and RBA10* right frontal areas (8 e 10 Broadman area), *RBA23* right posterior cingulum gyrus (23 Broadman area), *Aβ*_*42*_ β-amyloid42, *T-tau* total tau, *P-tau* phosphorylated tau, *MMSE* Mini-Mental State Examination, *RAVLT-I* Rey auditory verbal learning test - immediate recall, *RAVLT-D* Rey auditory verbal learning test - delayed recall, *T* time, *E* error

*p < 0.003 (Bonferroni corrected alpha). Only significant correlations are shown

### Longitudinal analysis

A subgroup of 12 OSA patients treated with beneficial CPAP therapy for a period of 12 months was included in the longitudinal analysis. In this subgroup, there were 10 men and 2 women, with a mean age of 68.64 ± 10.5. Patients showed an mean AHI of 42.00 ± 23.20 at baseline and of 4.48 ± 2.84 after 12 months of CPAP therapy (*p* = 0.003, *d* = 3.19). Baseline and longitudinal data of the OSA patient subgroup at the 12-month follow-up are presented in Table 5. No significant differences were found between the sample of patients undergoing follow-up analysis and the whole group of OSA patients evaluated at baseline in terms of demographic and clinical characteristics. Specifically, no differences were found for gender (*p* = 0.68), age (*p* = 0.66), CSF biomarkers (Aβ_42,_ *p* = 0.10; t-tau, *p* = 0.14; p-tau, *p* = 0.72), and MMSE (*p* = 0.96). In particular, considering CSF biomarkers analysis, the percentage of patients with pathological values was comparable between the two groups.

**Table 5:** Baseline and longitudinal data of OSA patient subgroup with a 12-month follow-up

	Baseline (mean ± SD)	12-montth follow-up (mean ± SD)	*p* value*
AHI	42.00 ± 22.20	4.47 ± 2.84	0.003
CSF data			
Aß_42_ (pg/mL) p.v. < 500 pg/mL	734.43 ± 271.65 (*n* = 2)		
t-tau (pg/mL) p.v. > 375 pg/mL	176.29 ± 122.63 (*n* = 2)		
p-tau (pg/mL) p.v. > 52 pg/mL	56.57 ± 66.430 (*n* = 1)		
Neuropsychological data			
MMSE (v.p. < 24)	26.17 ± 2.69 (< 24 *n* = 2)	27.58 ± 1.88 (< 24 *n* = 0)	NS
RAVLT-I (v.p. < 28.53)	30.32 ± 12.85 (< 28.53 *n* = 6)	30.92 ± 15.66 (< 28.53 *n* = 4)	NS
RAVLT-D (v.p. < 4.69)	6.68 ± 3.24 (< 4.69 *n* = 3)	7.08 ± 4.01 (< 4.69 *n* = 4)	NS
Raven (v.p. < 18.96)	25.90 ± 6.81 (< 18.96 *n* = 3)	27.33 ± 6.72 (< 18.96 *n* = 1)	NS
Stroop (T) (v.p. > 31.66)	47.25 ± 34.79 (> 31.66 *n* = 7)	31.26 ± 14.37 (> 31.66 *n* = 5)	0.002
Stroop (E) (v.p. > 2.83)	2.46 ± 3.22 (> 2.83 *n* = 4)	1.29 ± 1.74 (> 2.83 *n* = 2)	NS

*AHI* apnoea-hypopnoea index, *MMSE* Mini-Mental State Examination, *RAVLT-I* Rey auditory verbal learning test - immediate recall, *RAVLT-D* Rey auditory verbal learning test - delayed recall, *T* time, *E* error, *NS* non-significant

*Significant is set as *p* < 0.003 (Bonferroni corrected alpha)

**Fig. 3:**
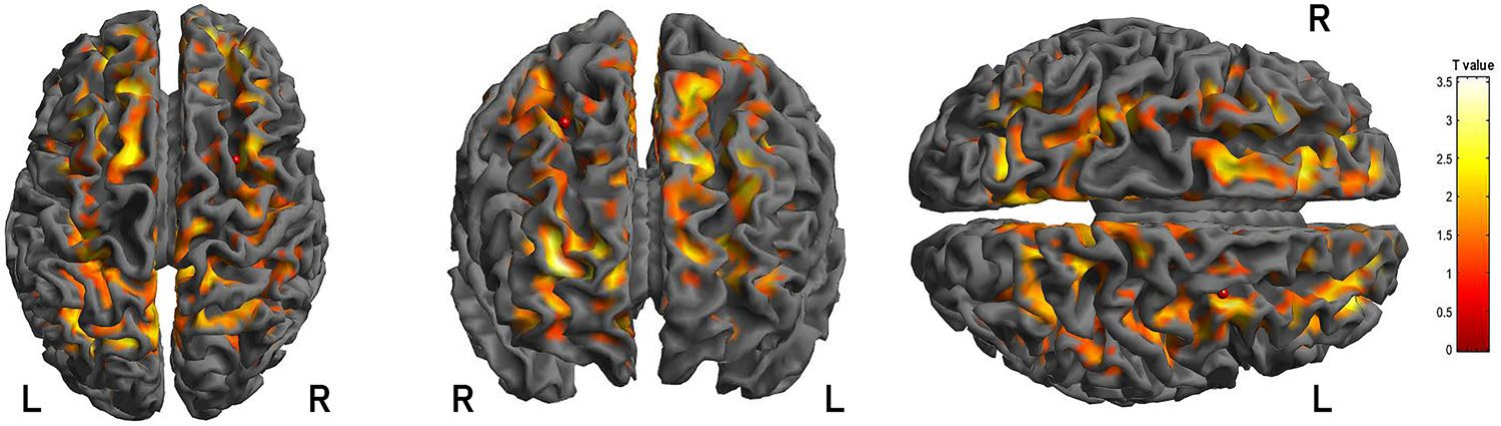
3D rendering showing the results of SPM comparisons between 18 F-FDG uptake in patients OSA patients undergoing the longitudinal evaluation. At the 12-month follow-up, a significant increase of cortical glucose consumption is detectable in right and left frontal and parietal cortex and in in OSA patients. R: right; L: left

Therefore, considering the group of patients undergoing the longitudinal analysis, the patients showed an improvement in the attention domain (Stroop [T]) (*p* = 0.002, *d* = 2.07) after 12 months of CPAP therapy. Moreover, a significant increase in ^18^F-FDG uptake in a wide cluster that involved the right and left frontal areas and left parietal lobe (BA 4, BA 6, BA 10) was also evident at the comparison with the baseline ^18^F-FDG PET data (see Table 6 and Fig. 3). No significant correlation was found between the AHI reduction between baseline and follow-up, cognitive performance, and the ^18^F-FDG uptake variation in the different BAs.

**Table 6:** Numerical results of SPM comparisons in the subgroup of OSA patients undergoing the longitudinal evaluation

Analysis	Cluster level					Voxel level		
	Cluster p(FWE-corr)	Cluster p(FDR-corr)	Cluster extent	Cortical region	Z score of maximum	Talairach coordinates	Cortical region	BA
Follow-up—Baseline	< 0.0001	< 0.0001	1603	R frontal	4.79	6,8,60	Superior frontal gyrus	6
				R frontal	4.69	30,12,54	Middle frontal gyrus	6
				R frontal	4.61	20,− 20,64	Precentral gyrus	6
	< 0.0001	< 0.0001	1590	L frontal	4.65	− 12,58,− 6	Superior frontal gyrus	10
				L frontal	4.62	− 24,40,20	Superior frontal gyrus	10
				R frontal	4.53	16,54,22	Superior frontal gyrus	10
	< 0.0001	< 0.0001	1294	R frontal	4.47	14,− 28,64	Precentral gyrus	4
				R frontal	4.35	− 36,− 14,46	Precentral gyrus	4
				L parietal	4.31	− 32,− 40,38	Supramarginal gyrus	40

In the “cluster level” section on the left, the number of voxels, the corrected P-value of significance, and the cortical region where the voxel is found, are all reported for each significant cluster. In the “voxel level” section, all of the correlation sites' coordinates (with the Z-score of the maximum correlation point), the corresponding cortical region, and BA are reported for each significant cluster. L, left; R, right; BA, Brodmann area. When the maximum correlation is achieved outside the grey matter, the nearest grey matter (within a range of 5 mm) is indicated by the corresponding BA

## Discussion

Several efforts have been made in the recent years to better understand the risk of sleep disorders, particularly OSA, as a potential contributor of neurodegenerative processes [61]. Although it has been documented that OSA can cause cognitive impairment, alteration in CSF AD biomarkers and reduction in cerebral ^18^F-FDG uptake [21–24], few studies have comprehensively evaluated all these biomarkers in middle-aged adult patients with OSA. Moreover, these studies investigated all the biomarkers at different times (from weeks to months) during the research work-up. Considering the importance of recognizing the increased risk of AD pathology in patients with OSA, the present study design was built to evaluate all these AD biomarkers in a very short period in order to check the relation among brain neurodegenerative processes, sleep quality and continuity, and cognitive performance in OSA patients. Finally, the longitudinal analysis represented a pilot evaluation to better understand the potential beneficial effects of a long period of CPAP treatment on AD biomarkers and cognition in patients with OSA (12 months—with respect to shorter follow-ups reported in previous investigations) [30–33]. Therefore, the present study focused on two aspects: on the one hand, to better define the effect of OSA on these biomarkers, it evaluated changes in different CSF, cognitive and ^18^F-FDG PET biomarkers in a large group of middle-aged adult patients with moderate to severe OSA compared to controls; on the other hand, to evaluate the possibility of improving cognitive performance and increasing brain function (measured by glucose consumption) in these patients, it explored the longitudinal effects of CPAP treatment on cognition and cerebral glucose consumption.

The main finding of the present study is the documentation of ^18^F-FDG PET uptake reduction in eloquent brain areas for AD pathology (also owing to the default mode network), such as the bilateral praecuneus, posterior cingulate cortex, and frontal areas, in OSA patients when compared to the CG. This finding concords with the very few previous PET studies including smaller groups of patients, which found a significantly reduced glucose consumption in the bilateral prefrontal areas, praecuneus, and left anterior cingulate cortex in OSA patients compared to controls [30–32]. Although the present study confirmed the impaired function of several brain areas in patients with OSA, the mechanisms at the basis of this malfunction have not been completely understood. For this reason, we also evaluated CSF AD biomarkers to test if this brain functional impairment can be due to pathological processes owing to AD pathology. Consistently, patients with OSA showed lower Aβ_42_ and higher p-tau proteins CSF levels compared to controls, and higher CSF tau proteins levels correlated with the reduction of brain glucose metabolism in a cluster of different brain frontal and parietal regions. Moreover, cognition was impaired in patients with OSA, in particular in the attention domain, which may be related to the observed pathological uptake of glucose in the frontal and temporal brain areas.

The main triggering events in OSA patients that may be responsible for these pathological modifications are nocturnal hypoxia and sleep fragmentation. Hypoxia is thought to be an important trigger in affecting brain glucose consumption in eloquent brain areas usually impaired at the beginning of AD pathology and associated with future cognitive impairment [62, 63]. Moreover, it has been suggested that sleep impairment due to OSA may induce preclinical AD biomarker changes, such as a reduction in CSF Aβ_42_ levels and an increase in brain amyloid plaque deposition [14–16]. Therefore, the CSF biomarker alterations in OSA patients may be explained by these two mechanisms: intermittent hypoxia, which has been hypothesized to alter brain amyloid metabolism inducing the activity of β-secretases, and sleep fragmentation, which may affect the glymphatic system [64–66].

Considering the CSF results, although the reduction of CSF Aβ_42_ levels in OSA patients has been already reported, the documentation of a significant increase in p-tau CSF levels is a novel finding with respect to the previous literature [15, 20]. The present finding may be due to the larger group of patients with OSA included in this study, compared to the past literature [15, 20]. Hence, these findings extend the previous literature on CSF AD biomarker changes in OSA patients and add evidence that p-tau protein alteration may reflect the presence of synaptic damage, neurodegeneration, and neuronal dysfunction, and the modification of brain glucose uptake documented by ^18^F-FDG PET as a result [67].

The second part of the present study describes the longitudinal analysis performed in a subgroup of OSA patients who were compliant to CPAP treatment. The positive effects of CPAP on cognitive performance and CSF AD biomarkers have been previously described [68, 69], and in this study, we confirmed not only the improvement in cognitive performance after long-term CPAP treatment, but also significantly documented the increase in cerebral glucose consumption in a wide cluster of areas, including the right and left frontal areas and the left parietal lobe. Notably, several areas improving glucose uptake take part to the default mode network, also with a central role. This finding reinforces the previously documented improvement in brain glucose metabolism in the bilateral precentral gyrus and left anterior cingulate cortex after a shorter period of CPAP treatment [30]. Consistently, beneficial CPAP treatment induces neural compensation and reduces cerebral dysfunction [30, 63, 70], thus improving brain glucose consumption and cognitive performance. Our findings, besides supporting previous longitudinal studies with a 3-month follow-up [30–33], broaden the previous knowledge through the documentation of the positive effects of long-term longitudinal CPAP treatment on both brain ^18^F-FDG PET and cognitive functioning, highlighting the clinical relevance of treating OSA by CPAP. Accordingly, recent evidence documented that the use of CPAP is associated with a lower risk of developing AD, dementia and mild cognitive impairment  in older adults [71]. Although the focus of the current study was the long-term effects of CPAP treatment, future studies should also consider the effect of behavioural interventions that an be concomitantly used in these patients, such as increasing physical activity, reducing alcohol consumption, or improving sleep hygiene.

The present study presents some limitations. The small sample size of patients recruited for the longitudinal analysis might have affected the statistical power of the results and prevented the generalisation of the longitudinal findings. In particular, the sample size of the subgroup of patients included in the longitudinal analysis was too small to perform further analysis in patients showing AD-typical biomarker changes at baseline. Another potential design limitation is that only patients compliant to CPAP treatment were included in the longitudinal analysis, which can reflect a selection bias of the study since those patients may be more likely those who experienced cognitive benefit. However, the novelty of these results invites future studies with a larger sample to further explore this aspect, possibly focusing on CSF biomarker values and on the cognitive trajectories of OSA patients treated by CPAP. Future studies may also explore if there is a dose-related response to CPAP therapy in terms of reduction of AHI or greater usage (e.g. 4.1 h vs 8 h of CPAP use a night) associated with greater improvement in cognitive performance. Another limitation is that sleep assessment through PSG was not repeated at follow-up in OSA patients; therefore, it is difficult to determine whether the changes in cognition and brain glucose consumption may be due to slow-wave sleep and REM sleep improvement and/or less sleep fragmentation. Further, sleepiness and fatigue were not assessed through self-reported scales, which raises the possibility that these factors may also account for the improvement of cognitive performance at follow-up. Although brain MRI was performed to exclude signs of brain atrophy and white matter abnormalities, a quantitative measure of brain atrophy was missing. Future studies should also consider measuring brain atrophy or white matter damage to assess whether OSA patients already demonstrate early signs of neurodegeneration, especially considering that both OSA and AD could have a bidirectional and cyclic potentiating effect on each other’s pathogenesis. For instance the progressive brain accumulation of amyloid plaques and intraneuronal neurofibrillary tangles of tau proteins in AD may determine changes in sleep patterns, even before dementia is recognized, thus potentially increasing the risk for OSA in AD. Finally, the CG did not constitute healthy volunteers and comprised of subjects with suspected malignancies undergoing ^18^F-FDG PET/CT and was found to be completely negative for various diseases. The lack of a PSG evaluation and a broad cognitive evaluation, as well as long-term follow-up in the CG is another limitation of the present study.

## Conclusion

Although increasing evidence shows pathological modifications of cognitive, CSF, and nuclear medicine biomarkers in OSA patients that may promote AD neurodegeneration [21–24], few studies have comprehensively assessed all these aspects. The present findings documented that patients with OSA show cerebral glucose consumption dysregulation, CSF AD biomarker alterations (both p-tau proteins and Aβ_42_) and cognitive impairment, thus highlighting the importance of increasing OSA screening and diagnosis in the middle-aged adult and elderly population, since CPAP treatment may improve cognitive performance and possibly restore brain functioning. Further studies are needed to evaluate the long-term effects of CPAP treatment to reinforce the hypothesis that AD biomarker changes in OSA conditions induced by hypoxia and sleep fragmentation can be considered reversible by CPAP, thus allowing the planning of preventive strategies against AD through CPAP treatment of OSA in middle-aged subjects presenting with this sleep disorder.

## Data Availability

The data underlying this article will be shared on reasonable request to the corresponding author.
